# Unexpected Pediatric Cluster of Enterovirus C105, Verona, Italy

**DOI:** 10.3390/v17020255

**Published:** 2025-02-13

**Authors:** Elena Pomari, Simone Malagò, Guglielmo Ferrari, Greta Romano, Antonio Mori, Andrea Matucci, Rebecca Feletti, Paolo Bonetti, Fausto Baldanti, Concetta Castilletti, Antonio Piralla

**Affiliations:** 1 Department of Infectious, Tropical and Microbiology, IRCCS Sacro Cuore Don Calabria Hospital, 37024 Negrar di Valpolicella, Verona, Italy; elena.pomari@sacrocuore.it (E.P.); simone.malago@sacrocuore.it (S.M.); antonio.mori@sacrocuore.it (A.M.); andrea.matucci@sacrocuore.it (A.M.); rebecca.feletti@sacrocuore.it (R.F.); 2 Microbiology and Virology Department, Fondazione IRCCS Policlinico San Matteo, 27100 Pavia, Italy; guglielmo.ferrari01@universitadipavia.it (G.F.); greta.romano01@universitadipavia.it (G.R.); fausto.baldanti@unipv.it (F.B.); antonio.piralla@unipv.it (A.P.); 3 Pediatric Clinic, IRCCS Sacro Cuore Don Calabria Hospital, 37024 Negrar di Valpolicella, Verona, Italy; paolo.bonetti@sacrocuore.it; 4 Department of Clinical, Surgical, Diagnostic and Pediatric Sciences, University of Pavia, 27100 Pavia, Italy

**Keywords:** enterovirus, rhinovirus, enterovirus-C105, RNA, Sanger sequencing, whole-genome sequencing

## Abstract

In an epidemiologic investigation of Enterovirus (EV) infections in a Verona hospital, September 2022–September 2024, we detected EV-C105 in six pediatric patients with upper respiratory symptoms between March and May 2023. The primary objective was to describe the local incidence of EV cases. The secondary objective was to perform Sanger’s genomic characterization and the whole-genome sequencing (WGS) of EV-C105. The proportion of positive EV results was calculated based on routine molecular method testing. An available cohort of 114 underwent Sanger sequencing, and the six EV-C105 were characterized with WGS. Overall, 96% EV results were from the upper respiratory tract. The total proportion of positives in children was 83%. Out of the typed 114, 90% were Rhinoviruses and 9%, EVs. Notably, six pediatric cases were EV-C105, placing together in a unique cluster with 99% of nucleotides belonging to the European lineage with the highest Average Nucleotide Identity, including EV-C104, EV-C109, and EV-C118. Our data describes the first cluster indicating that EV-C105 incidence may be higher than previously estimated. However, a limitation for affirming this hypothesis is the lack of a more in-depth epidemiological investigation on a larger case series with the possibility of including data from coordinated laboratories.

## 1. Introduction

The viral genus *Enterovirus* (EV) belongs to the family *Picornaviridae* and can cause a range of acute symptoms, from mild common colds to severe systemic infections such as meningitis, myocarditis, and flaccid paralysis [1]. EV infections can spread mainly through the fecal–oral route as well as through respiratory secretions from infected individuals. Although enteroviral infections can also affect adults, newborns and infants are at the highest risk for developing severe conditions [1]. Worldwide, an estimated 1 billion or more human enteroviral infections occur annually. In recent decades, there have been alarming epidemics of non-polio EVs (e.g., EV-D68, EV-A71) [2], and after COVID-19 and monkeypox, another scary menace is poliovirus (a subtype of EV species C that still circulates with potential risk of causing poliomyelitis) [3]. Thus, there is a pressing need for coordinated monitoring and preparedness efforts against EVs. At present, non-polio EV surveillance has not been implemented in Italy or in most European countries, and data are currently collected by national reports and single studies [2,4]. Among the non-polio EVs, EV-C105 is a species C genotype that was identified for the first time in 2010 in the Democratic Republic of the Congo (strain 34S) and linked to acute flaccid paralysis [5]. In particular, current monitoring data suggest that EV-C105 has a global distribution, although the overall incidence appears to be very low. The present work summarizes the EV’s genus epidemiologic surveillance data at IRCCS Sacro Cuore Don Calabria Hospital (SCDC) and reports for the first time a cluster of EV-C105 circulating in patients in Verona province (Veneto Region), Northeast Italy.

## 2. Material and Methods

### 2.1. Study Design

A laboratory-based epidemiologic investigation was started at the SCDC after the COVID-19 pandemic, and basic data on specimens positive for Enterovirus/Rhinovirus, including genotype, have been prospectively collected, covering the Verona province area. A total of 658 EV/Rhinovirus (RV) cases were detected through routine molecular testing (Table S1) from September 2022 to September 2024 at the SCDC. Routine molecular testing was performed using a Biofire^®^FilmArray^®^ pneumonia plus bioMérieux (FilmPP) panel or respiratory panel amplification (Anatolia Geneworks) (Anatolia) for the upper respiratory tract, a Biofire^®^FilmArray^®^ RP21+ panel (bioMérieux) (FilmRP) for the lower respiratory tract, and a Biofire^®^FilmArray^®^ Meningitis/Encephalitis (bioMérieux) (FilmME) panel for cerebrospinal fluid (CSF). A descriptive analysis was conducted in order to obtain proportions of positivity based on age and sex. For the purpose of this study, no association analysis (i.e., demographic, clinical, diagnostic methods) was conducted. Sanger sequencing was performed at the SCDC for typing available samples, and the EV-C105 whole-genome characterization was conducted at the Microbiology and Virology Department of Fondazione IRCCS Policlinico San Matteo (Pavia, Italy).

### 2.2. Sanger and Whole-Genome Sequencing

An available subset of specimens (*n* = 114) underwent Sanger sequencing for the typingof the VP4-VP2 region, as previously reported [6]. The EV-C105s were also characterized for the partial VP1 region following the WHO protocol [7], and a maximum likelihood (ML) phylogenetic tree was constructed using IQ-TREE multicore version 2.3.3. The robustness of branches was evaluated using ultrafast bootstrap approximation tests. The whole-genome sequence of EV-C105 strains was also recovered using the metagenomic approach, as previously described [8,9]. The IDseq pipeline was used to perform read quality control, alignment, and assembly to derive consensus EV-C105 whole-genome sequences by exploiting the metagenomic pipeline (reference genome: KM880100.1).

## 3. Results

### 3.1. Description of Total Enterovirus Cohort

**Figure 1:**
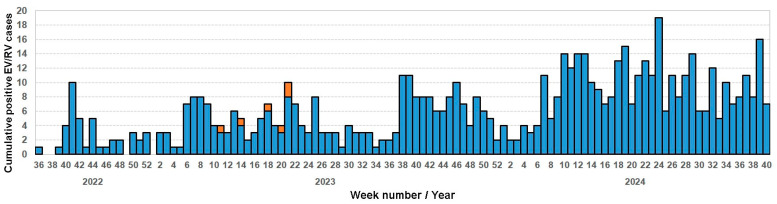
Weekly distribution of the 208 cumulative positive EV/RV cases in 2022 and 2024. EV-C105s are identified in orange.

The majority of EV/RV cases (633/658; 96%) were from the upper respiratory tract, 23/658 (3.5%) from the lower respiratory tract, and 2/658 (<1%) from CSF (Table S1). Among the total, 56% (367/658) of samples were collected from males. The total proportion of positives in children (<18 years) was 83% (545/658), and the remaining 17% (113/658) were adult-aged patients up to 101 years old. Generally, EV positivity in the low respiratory tract sample and CSF was detected in younger adults and adults. Regarding EV circulation, no peaks were observed during the study period; however, a general increased number of EV cases was observed in 2024 (Figure 1), probably due to a contextual increase in testing. In the available 114 samples, 90% (103/114) were RVs (n = 57 RV-A, n = 14 RV-B, n = 32 RV-C) and 9% (11/114) were EVs (n = 1 Coxsackievirus CV-A10, n = 1 CV-B2, n = 6 EV-C105, n = 1 EV-C118, n = 2 EV-D68).

### 3.2. Sanger and Whole-Genome Characterization of EV-C105

**Figure 2:**
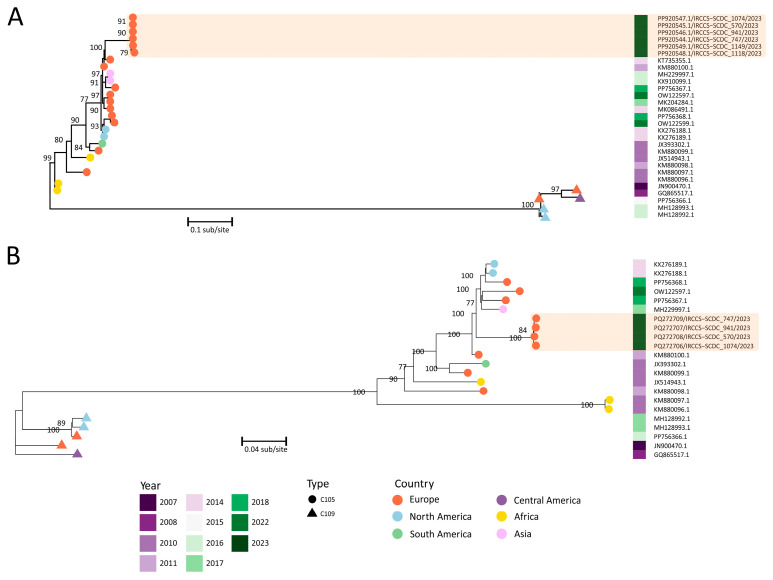
Phylogenetic trees based on partial VP1 (**A**) and whole-genome nucleotide sequences (**B**) with all available Enterovirus C105 genome sequences. The heatmap indicates the sampling date (2007–2023), while each node is colored according to the origin continent (Europe, Northern America, Southern America, Africa, Asia). The robustness of the branches is shown on the label nodes with bootstrap values (bootstrap support cutoff, ≥70%). The units of branch length are the number of substitutions divided by the length of the sequence. Enterovirus C109 sequences were used as outgroups.

The EV-C105 samples were first sequenced for the partial VP1 region using the Sanger method. Phylogenetic analysis (using 18 available VP1 EV-C105s from the NCBI and with EV-C109 as the outgroup) showed that they were all placed together in a cluster sharing 99% of nucleotides and belonging to the European lineage (Figure 2A). Compared to the reference prototype (EV-C105 strain 34S, JX514943), BLAST analysis showed that the sequences have a range of 90–89% identity. Whole-genome sequencing was also performed. Four out of six samples contained enough read coverage to recover consensus sequences (sample 1074: coverage depth 43.3X and breadth 99.7%; sample 941: 304.4X and 100%; sample 570: 181.9X and 97.9%; sample 747: 93X and 98.1%). The Average Nucleotide Identity (ANI) was determined by consensus for similarity in terms of nucleotide identity with a mean of 92.3% (range: 84.6−100), confirming a high similarity. A phylogenetic analysis was performed with all available EV-C105 strains (n = 13) from the NCBI, using EV-C109 as the outgroup. The analysis confirmed that the EV-C105 Italian strains belonged to the same European cluster (strains collected in the period 2011–2022), as the VP1 analysis showed (Figure 2A). The EVs’ whole-genome sequences were downloaded from the NCBI (n = 87) and most were classified as EV-C104 (23) and EV-C105 (18); the remaining were typed as EV-C109 (5), EV-C117 (10), EV-C118 (4), EV-C96 (2), EV-C99 (1), CVA (7), and Poliovirus (17). A phylogenetic tree based on these sequences was drawn using Poliovirus as the outgroup (Figure S1). Clear species-based clusters can be seen closely together in the tree (Figure 2B). Moreover, EV-C105 showed the highest ANI with EV-C104 (ANI: 86.7%; range: 83.9–90), EV-C109 (ANI: 82.7%; range: 80.8–84), and EV-C118 (ANI: 82.7%; range: 80–84.2) being greater than EV-C117 (ANI: 78.5%; range: 75.9–85).

## 4. Discussion

The introduction of the molecular typing surveillance of EV promoted the identification of new EVs, especially within EV species C, where in the last 15 years, several new genotypes were discovered (EV-C104, -C105, -C109, -C117, -C118). Among these new viruses, EV-C105 is an emerging neurotrophic enterovirus firstly identified in 2010 in the Republic of the Congo (strain 34S) in the stool of a human with fatal acute flaccid paralysis [5]. So far, only 24 sequences have been deposited in a public database. This suggests a low circulation of EV-C105 but also a possible underestimation of the number of cases due to the lack of typing surveillance programs. During our EV surveillance, we detected six cases of EV-C105 in a short period of time, suggesting an unrevealed circulation of this virus in Verona province. Five patients resided in the northwest area with a perimeter of 32 km and an area of 70 km^2^; however, no evidence of linkage for all the EV-C105 cases observed here was established. The phylogenetic analysis showed the genetic relationship among the EV-C105 strains described in the last 15 years in Italy in Pavia (KM880098 and KM880100) and Ancona (MK204284). Moreover, most of the previous studies have reported few cases or individual cases [4,10,11,12], whereas our data indicate a first cluster indicating that EV-C105 incidence may be higher than previously estimated. However, a limitation for affirming this hypothesis is the lack of a more in-depth epidemiological investigation on a larger case series with the possibility of including data from coordinated laboratories. In our series, EV-C105 infections were associated only with upper respiratory symptoms, whilst previous studies showed how EV-C105 was responsible for signs or symptoms of lower respiratory tract, gastrointestinal, and central nervous system infections. None of our patients reported severe complications (and no follow-up was available), and the virus detection in all of them suggests a potential pathogenic role of viral co-infection, as is already known for the other viruses detected. However, due to the low numbers of EV-C105 cases, this did not allow us to draw major conclusions on the role of EV-C105 as a major threat to human health, especially in younger ages. To conclude, our findings provide new insights into the circulation of human EV-C105 in Verona province (Veneto Region, Northeast Italy), showing the circulation of this genotype. Moreover, our data join other works in the literature that show how important the continuous monitoring of EVs is [2,4], as well as the effort to implement data collection between coordinated laboratories, especially with a focus on the evolutionary study given, e.g., by the high genetic variability of such viruses, as shown by recent works [8,13], on recombination events.

## Data Availability

Amplicon sequences were submitted to GenBank, accession number range: PP920544-PP920549. Whole-genome sequences were submitted to GenBank, accession number range: PQ272706-PQ272709.

## Supplementary Materials

The following supporting information can be downloaded at: https://www.mdpi.com/article/10.3390/v17020255/s1, Figure S1: Maximum Likelihood (ML) tree based on whole genome sequences of Enterovirus C species (EVC104, EVC105, EVC109, EVC117, EVC118, Coxsackievirus A, EVC96, EVC99, Po-liovirus). The color of tree nodes classify strains typing (C104, C105, C109, C117, C118, CVA, C96, C99, PV). GenBank accession number is reported followed by an inner layer containing sampling date (1975–2023). The outer layer represents the origin continent (Europe, Asia, North America, South America, Africa), followed by country names (third layer). Poliovirus sequences were used as outgroups; Table S1: Age and gender descriptive table of positive results recorded from 1 September 2022 to 30 September 2024 for human rhinovirus/enterovirus detected with Biofire^®^FilmArray^®^ pneumonia plus (FilmPP) (BioMérieux), Biofire^®^FilmArray^®^ RP21+ panel (FilmRP) (BioMérieux), Biofire^®^FilmArray^®^ Meningitis/Encephalitis (FilmME) panel (BioMérieux) or respiratory Panel amplification Anatolia Geneworks (Anatolia).
