# Supplementary material for: Unexpected Pediatric Cluster of Enterovirus C105, Verona, Italy
Source: Viruses. 2025 Feb 13;17(2):255. doi: 10.3390/v17020255 (PMC11861629; doi:10.3390/v17020255)
Supplement: Supplementary file 1 [file viruses-17-00255-s001.zip › Table S1.pdf]

Table S1. Age and gender descriptive table of positive results recorded from 1st September 2022 to 30th September 2024 for human rhinovirus/enterovirus detected with Biofire®FilmArray® pneumonia plus (FilmPP) (BioMérieux), Biofire®FilmArray® *RP21+* panel (FilmRP) (BioMérieux), Biofire®FilmArray® Meningitis/Encephalitis (FilmME) panel (BioMérieux) or respiratory Panel amplification Anatolia Geneworks (Anatolia).

Assay	Gender	N	Age (years)			
			Mean	Median	Minimum	Maximum
FilmRP	M	344	10.6	3.00	0.00	95.0
	F	269	11.3	3.00	0.00	101.0
Anatolia	M	12	16.33	3.00	0.00	82.0
	F	8	36.63	34.00	1.00	94.0
FilmPP	M	14	69.79	69.50	55.00	91.0
	F	9	60.00	64.00	25.00	86.0
FilmME	M	2	34.00	34.00	31.00	37.0
