# Supplementary figures and images for: Unexpected Pediatric Cluster of Enterovirus C105, Verona, Italy
Source: Viruses. 2025 Feb 13;17(2):255. doi: 10.3390/v17020255 (PMC11861629; doi:10.3390/v17020255)

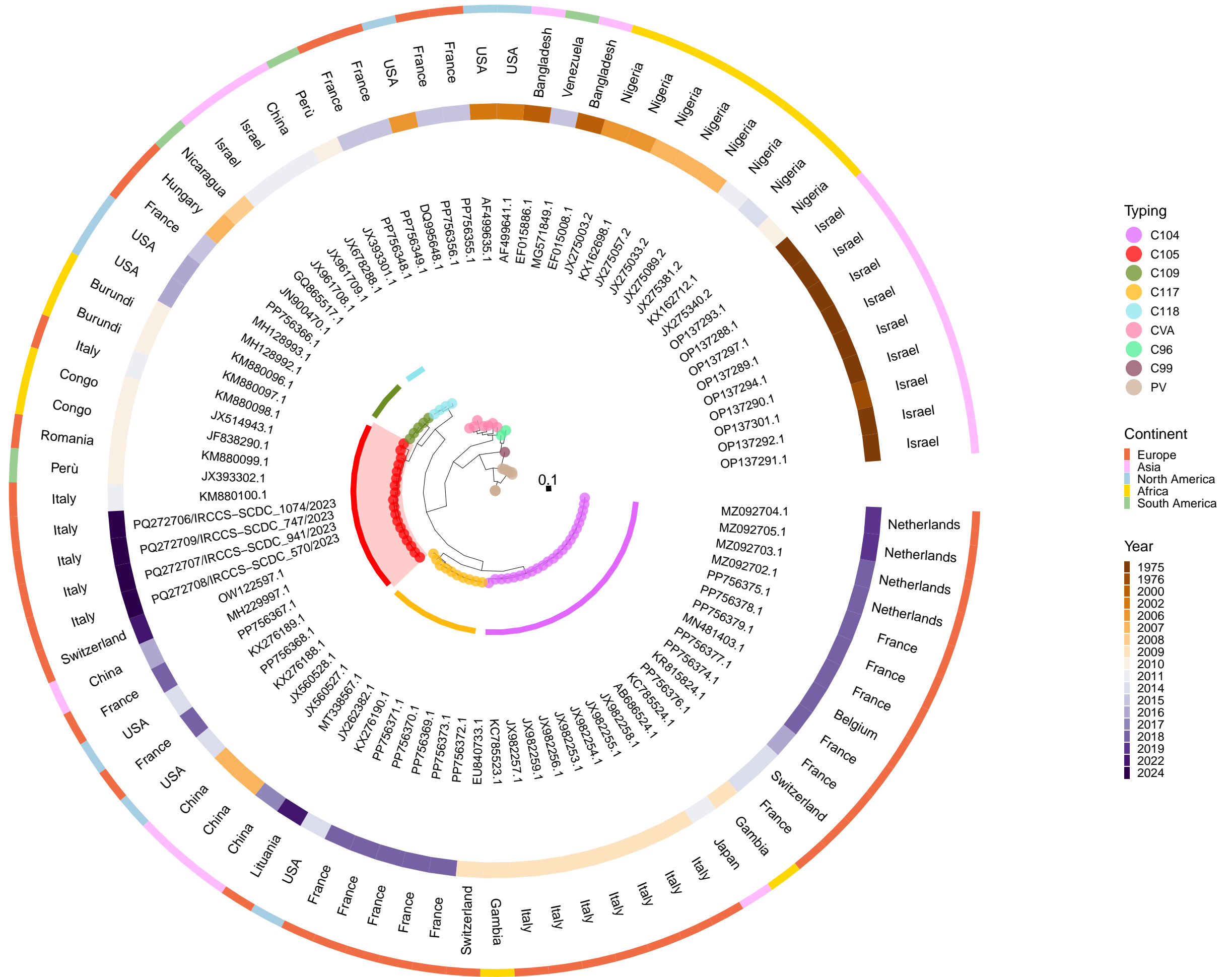

Supplement: Supplementary file 1 [file viruses-17-00255-s001.zip › Figure S1.pdf]
